# A decellularized flowable placental connective tissue matrix supports cellular functions of human tenocytes in vitro

**DOI:** 10.1186/s40634-022-00509-4

**Published:** 2022-07-18

**Authors:** Yong Mao, Nikita John, Nicole M. Protzman, Adam Kuehn, Desiree Long, Raja Sivalenka, Radoslaw A. Junka, Anna Gosiewska, Robert J. Hariri, Stephen A. Brigido

**Affiliations:** 1 Laboratory for Biomaterials Research, Department of Chemistry and Chemical Biology, Rutgers University, 145 Bevier Rd., Piscataway, NJ 08854 USA; 2 Healthcare Analytics, LLC, 78 Morningside Dr., Easton, PA 18045 USA; 3 Research & Development, Degenerative Diseases, Celularity Inc., 170 Park Ave., Florham Park, NJ 07932 USA

**Keywords:** Biomaterials, Extracellular matrix, Flowable, Inflammatory response, Injectable scaffold, Placenta, Tendon healing, Tenocytes, Tissue particulates

## Abstract

**Purpose:**

Injectable connective tissue matrices (CTMs) may promote tendon healing, given their minimally invasive properties, structural and biochemical extracellular matrix components, and capacity to fill irregular spaces. The purpose of this study is to evaluate the effects of placental CTMs on the cellular activities of human tenocytes. Decellularization, the removal of cells, cell fragments, and DNA from CTMs, has been shown to reduce the host’s inflammatory response. Therefore, the authors hypothesize that a decellularized CTM will provide a more cell-friendly matrix to support tenocyte functions.

**Methods:**

Three human placental CTMs were selected for comparison: AmnioFill® (A-CTM), a minimally manipulated, non-viable cellular particulate, BioRenew™ (B-CTM), a liquid matrix, and Interfyl® (I-CTM), a decellularized flowable particulate. Adhesion and proliferation were evaluated using cell viability assays and tenocyte migration using a transwell migration assay. Gene expression of tenocyte markers, cytokines, growth factors, and matrix metalloprotease (MMP) in tenocytes were assessed using quantitative polymerase chain reaction.

**Results:**

Although A-CTM supported more tenocyte adhesion, I-CTM promoted significantly more tenocyte proliferation compared with A-CTM and B-CTM. Unlike A-CTM, tenocyte migration was higher in I-CTM than the control. The presence of I-CTM also prevented the loss of tenocyte phenotype, attenuated the expression of pro-inflammatory cytokines, growth factors, and MMP, and promoted the expression of antifibrotic growth factor, *TGFβ3*.

**Conclusion:**

Compared with A-CTM and B-CTM, I-CTM interacted more favorably with human tenocytes in vitro. I-CTM supported tenocyte proliferation with reduced de-differentiation and attenuation of the inflammatory response, suggesting that I-CTM may support tendon healing and regeneration in vivo.

**Supplementary Information:**

The online version contains supplementary material available at 10.1186/s40634-022-00509-4.

## Background

Tendon healing is a slow and complex process that cannot restore the structure and function of the native tendon, owing to the dedifferentiation of tenocytes, an influx of inflammatory factors, and the formation of scar tissue. The loss of mechanical competence is largely attributed to a disorganized extracellular matrix (ECM) and misaligned fibers [37]. As a result, the healed tendon is at an increased risk of tendon degeneration and re-rupture [12]. Therefore, new methods have been suggested to enhance tendon repair and regeneration [46], including the use of exogenous scaffolds [3, 14, 25], and more recently, flowable connective tissue matrices (CTMs) [8, 11, 51].

Tenocytes are resident cells of the tendon that modulate the ECM in response to mechanical loading [4] and are one of the major cell types involved in tendon repair and regeneration. The direct interaction between tenocytes and flowable CTMs has the potential to influence tendon healing. For this reason, an ideal scaffold for tendon regeneration has been defined as an ECM supporting cell-material interactions, cell adhesion, proliferation, migration, and differentiation, while minimizing the immune response [8, 40, 46].

Loss of tenocyte phenotype has been hypothesized to result in the formation of scar tissue [29] and is associated with a decrease in the expression of tenocyte markers, including scleraxis (*SCX*) [31, 43], tenomodulin (*TNMD*) [21, 44], tenascin-C (*TNC*) [38], type I collagen (*COL1A1*) [10, 42], type III collagen (*COL3A1*) [10, 42], and decorin (*DCN*) [39] (Table 1). Previous research has demonstrated that the introduction of an ECM can reduce the dedifferentiation of primary cells [27, 53].

**Table 1:** Markers of tenocyte phenotype. Tenocyte phenotypic markers, their associated functions, and references are provided

Marker	Function(s)	Reference(s)
Scleraxis (*SCX*)	Transcriptional activator that regulates the expression of tenomodulin and the proliferation of tenocytes	[31, 43]
Tenomodulin (*TNMD*)	A transmembrane glycoprotein, a specific marker of mature tendons and ligaments that has anti-angiogenic activity and promotes the maturation and maintenance of the differentiated tendon phenotype	[21, 44]
Tenascin-C (*TNC*)	A glycoprotein known to be present in healthy tendon and is involved in the regulation of collagen fibrillogenesis	[38]
Type I Collagen (*COL1A1*)	The most abundant molecule in the tendon extracellular matrix	[10, 42]
Type III Collagen (*COL3A1*)	The 2nd most abundant collagen in tendon, and its expression is stimulated during tendon repair	[10, 42]
Decorin (*DCN*)	Necessary for maintaining collagen fibril integrity in mature tendon	[39]

Aberrant inflammation impairs the rate of tendon healing and increases deposition of scar tissue, resulting in a weaker tendon [33]. Tenocytes have been reported to produce and respond to pro-inflammatory cytokines such as interleukin-8 (IL-8), interleukin-1 beta (IL-1β), and tumor necrosis factor alpha (TNF-α) [17] and to regulate the production of inflammation modulator and pro-fibrotic factor transforming growth factor beta-1 (TGF-β1) as well as anti-fibrotic factor, transforming growth factor beta-3 (TGF-β3) [18, 32, 36, 49]. Increased production of inflammatory cytokines, such as IL-1β, activates some matrix metalloproteinases, such as matrix metalloproteinase 1 (MMP-1), which promotes the degradation of the ECM [48]. Extracellular matrices, however, have been shown to interact with tenocytes and modulate inflammatory gene expression to support tendon healing [30].

A variety of flowable CTMs are now commercially available for the augmentation of tendon repair [2, 8, 20, 22, 23, 35]. Human placental CTMs represent a unique subset of biological scaffold particulates, given the inherent structural, biochemical, and immunogenic properties of placental tissue [23, 35]. During the tendon healing process, placental tissue CTMs have the potential to reduce dedifferentiation, lessen inflammation, limit tissue adhesion formation, and accelerate the healing process.

Placental CTMs, like all implanted materials, evoke a host foreign body reaction. The extent of the reaction is correlated with the quantity of DNA in the ECM product [19, 41]. To limit the host inflammatory immune response, some placental CTMs undergo the process of decellularization [1, 13]. Decellularized CTM biomaterials have been suggested as a means of limiting the host inflammatory immune response, improving integration with host issues, and promoting more rapid and complete healing [1, 8].

In recent years, flowable CTMs have gained increased attention. Like non-flowable scaffolds, flowable CTMs provide structural and biochemical ECM components [7, 8]. In contrast with their non-flowable counterparts, flowable CTMs offer the added benefits of being minimally invasive with the capacity to fill irregular spaces [7, 8]. However, the direct interaction between tenocytes and flowable placental CTMs has not yet been analyzed. Therefore, we evaluated the effects of flowable particulated placental CTMs on the cellular activities of human tenocytes. Based on direct comparison with two non-decellularized placental CTMs, we hypothesize that a flowable decellularized placental CTM will provide a more cell-friendly matrix to support tenocyte adhesion, proliferation, migration, phenotype maintenance, and inflammatory response.

## Methods

### CTMs

Three CTMs were selected: AmnioFill® Placental Tissue Allograft (A-CTM) (MiMedx Group, Inc., Marietta, GA), BioRenew™ Placental Tissue Matrix Therapy (B-CTM) (SKYE Biologics, El Segundo, CA), and Interfyl® Human Connective Tissue Matrix (I-CTM) (Celularity, Inc., Florham Park, NJ). Samples were stored at ambient temperature. All three CTMs are processed from human tissue according to the American Association of Tissue Banks (AATB) standards, and are regulated as a human cell, tissue, or cellular or tissue-based product (HCT/P) under Section 361 of the Public Health Service Act and 21 CFR Part 1271.

A-CTM is marketed as a minimally manipulated, non-viable cellular tissue matrix allograft that contains multiple ECM proteins, growth factors, cytokines, and other specialty proteins present in placental tissue to help enhance healing [22, 23]. SMR^2^T_TM_ Technology and patented PURION® processing are used to dehydrate and sterilize the tissue, while preserving the ECM. According to the manufacturer, no chemicals are used in the PURION® process that might result in chemical cross-linking or decellularization. The tissue is also terminally sterilized for an additional level of safety.

B-CTM tissue was obtained from SKYE Biologics. B-CTM is marketed as an all-natural treatment that utilizes powerful growth factors, collagens, ECM proteins, and bioactive molecules naturally found in human placental tissues to replace the body’s missing CTM. The HCT/P is processed in a controlled environment using methods designed to prevent contamination of tissues. It is exposed to antibiotics during initial processing, and subsequently subjected to multiple rinse steps using sterile saline. Final products are sized and packaged and are terminally sterilized by E-Beam irradiation technology.

I-CTM is an allogenic decellularized particulate human placental CTM consisting of natural human structural and biochemical ECM components [8]. I-CTM is intended for use as the replacement or supplementation of damaged or inadequate integumental tissue. I-CTM is minimally manipulated during processing and is marketed in accordance with the FDA’s requirements for HCT/Ps. I-CTM is produced from donated placentas, after normal, healthy, full-term pregnancy. The tissue is washed and scraped to remove extraneous tissues and cells. The tissue is then decellularized using an osmotic shock followed by a mild detergent treatment, dried, and terminally sterilized with e-beam irradiation.

The testing materials are commercially available products. Therefore, it has been determined that this research does not require Institutional Review Board approval, since there is no intervention or interaction with human subjects.

### Primary cells

Human tenocytes were purchased from ZenBio Inc. (Research Triangle, NC). Cells were cultured in tenocyte medium (ZenBio Inc.), following the manufacturer’s instructions.

### Preparation of solutions of CTMs for coating

B-CTM samples were provided as a solution containing placental tissue matrix. To measure the concentration of the CTM, B-CTM solutions were centrifuged at 10,000 rpm in a desktop microfuge (Eppendorf, Enfield, CT) for 5 min. The supernatant was collected and saved, and the pellet was lyophilized using a lyophilizer (FreeZone, Labconco Corporation, Kansas City, MO). The dried mass of CTM was weighed and resuspended in the original, saved supernatant to 12.5 mg/mL. A-CTM samples are fibrous particulates. Particulates were weighed and resuspended in dH_2_O at 12.5 mg/mL. I-CTM particulates were weighed and resuspended in dH_2_O at 12.5 mg/mL.

### Coating of CTMs on ultra-low attachment plates

0.2 mL/well of solutions containing CTMs was added to the wells of ultra-low attachment plates (ULAPs) (Cell-Repellent 48-Well Microplate, Greiner Bio-One, Monroe, NC). The plates were open and left to air-dry in a convention hood for 2–3 days at room temperature. The coatings were sterilized under ultraviolet light in a biosafety cabinet for 1 hour (h) before use.

### Assessment of cell adhesion to CTMs

Before cell seeding, the wells coated with CTM were gently rinsed with 0.5 mL/well of phosphate buffered saline (PBS) once. Human tenocyte cells at passage 4 (P4) were cultured to 80% confluence in 10 cm cell culture dishes following the manufacturer’s instruction. Cells were rinsed with 5 mL PBS/dish once. One milliliter of 0.25% trypsin (Gibco®, Thermo Fisher Scientific, Waltham, MA) was added to each dish and incubated at 37 °C for 5 min. Two milliliters of culture medium was added to the dish to neutralize the trypsin. Cells were transferred to 15 mL conical tubes and centrifuged at 1000 rpm for 5 min. Cells were resuspended in complete growth medium and cell numbers were quantified using a hemocytometer.

2 × 10^4^ tenocytes were added per well (2.6 mg/cm^2^) of a 48-well tissue culture polystyrene plate (TCP) coated with CTM. As a control, cells were added to wells of 48-well TCP. Plates were incubated at 37 °C with 5% CO_2_ and 95% humidity. After incubation for 24 h, medium was removed. The number of adhered cells was detected using alamarBlue assay, a metabolic activity assay. Briefly, 0.2 mL/well of alamarBlue solution (complete growth medium+ 10% alamarBlue reagent) (Bio-Rad Laboratories, Philadelphia, PA) was added to each well and incubated at 37 °C for 30 min. After incubation, 0.1 mL/well of supernatant was transferred to a 96-well plate. Fluorescent intensity was read using a multimode microplate reader (Spark®, TECAN, Switzerland) at excitation/emission (Ex/Em) equal to 540 nm/590 nm. Fluorescent intensity was expressed in arbitrary units (AU). To convert the fluorescent intensity to number of cells, a standard curve was determined, as follows. Tenocytes were seeded onto TCP at different densities (serially diluted (1:2) from 40,000 cells/well to 313 cells/well) and cultured for 24 h. A linear curve was obtained by graphing cell viability of fluorescent intensity (AU) and cell number (*R*^2^ > 0.99, data not shown). The equation of this curve was used to convert the fluorescent intensities (AU) to the numbers of adherent cells for testing samples.

### Assessment of cell viability on CTMs

2 × 10^4^/well of tenocytes were added to 48-well plates coated with CTM. As a control, cells were added to wells of 48-well TCP. Four sets of plates were set up and incubated at 37 °C with 5% CO_2_ and 95% humidity for 1, 2, 4, and 7 days, respectively. At the first time point, medium was removed from each well of all plates and fresh medium was added. The number of viable cells in the first set of plates was measured using alamarBlue assay. Fresh medium was added to the second, third, and fourth set of plates and continued to culture at 37 °C. At the second, third, and fourth time point, the viability of cells of each set was measured using alamarBlue assay.

### Live staining of cells

Tenocytes were cultured on the CTMs, and the medium was changed every 3 days. On Day 7, the medium was removed from each well, 0.2 mL/well of fresh complete growth medium containing 50 nM Calcein AM (C1430, Thermo Fisher Scientific, Waltham, MA) was added to each well. After incubation for 30 min at 37 °C, the medium was removed. Cells were washed twice with PBS and observed under epi-fluorescent microscope (Zeiss Axio Observer D1, Jena, Germany).

### Immunofluorescent staining of cells

To detect the protein level of type I collagen (Col 1) in cells on Day 7, immunofluorescent staining was performed, as previously described [28]. Briefly, tenocytes were fixed with 4% paraformaldehyde for 1 h and permeabilized in 0.5% Triton X100 in PBS for 1 h. The fixed and permeabilized cells were stained with anti-Col 1 antibody (pAb ab34710, Abcam, Waltham, MA) overnight, followed by secondary goat anti-rabbit IgG-Alexa 555 antibody (Life Technology, Carlsbad, CA) for 1 h. Nuclei were stained with Hoechst dye 33258 (Thermo Fisher Scientific, Waltham, MA). Stained samples were imaged under epi-fluorescent microscope (Zeiss Axio Observer D1).

### Conditioned media for migration assay

2 × 10^4^/well of tenocytes were seeded onto CTM-coated wells. After culturing for 24 h, the medium was removed. 0.4 mL/well of migration medium (complete culture medium diluted 1:1 with DMEM base medium to reduce FBS concentration to 5%) was added to each well and incubated at 37 °C for 24 h. The supernatants (24-h conditioned media) were collected from each well and used immediately for the migration assay.

### Transwell migration assay

0.4 mL of conditioned medium was transferred (collected as described above) to each well of a 24-well plate. A transwell insert (6.5 mm diameter inserts, 8.0 μm pore size PET membrane, Costar, Corning, NY) was placed into each well, containing conditioned medium. Tenocytes were resuspended at 3 × 10^5^/mL in DMEM base medium (no FBS). 0.3 mL of tenocytes (0.9 × 10^5^) were added to each transwell insert and incubated at 37 °C with 5% CO_2_ and 95% humidity. To determine total cell number (control), 0.3 mL/well of tenocytes were added to wells of a 24-well TCP plate and incubated with the plate containing transwells. After incubation for 24 h, the inserts were transferred to a new 24-well plate, containing 0.4 mL/well MTT solution (MTT (3-(4,5-Dimethylthiazol-2-yl)-2,5-Diphenyltetrazolium Bromide, M6494, Thermo Fisher Scientific, Waltham, MA)) (1 mg/mL in DMEM base medium), and the MTT solution was added to the control wells and incubated for 1 h. The inserts were washed in PBS three times and the stain inside of each insert was carefully removed, using cotton swabs. Each insert was immersed in 200 μL/well of dimethyl sulfoxide (DMSO) in a fresh 24-well plate. The plate was rocked to mix and extract the purple colored MTT-formazan. Controls stained with MTT were washed three times with PBS and extracted with 200 μL/well of DMSO. One hundred microliters of DMSO extractants were transferred to a well of 96-well plate and the absorbance was read at 570 nm (100 μL of pure DMSO was served as blank and 650 nm as reference) using a multimode microplate reader (Spark®, TECAN, Switzerland). Migration is expressed as the percentage of migrated cells to the total number of cells (OD_sample_/OD_control_ × 100%).

### Preparation of RNA lysates for assessment of phenotype maintenance

Tenocytes at 2 × 10^4^/well were added to each well of 48-well plates coated with CTM. Two sets of samples were set up. After culturing for 24 h, the medium was removed from all samples. Fresh culture medium was added to each well and incubation continued. After 24 h, the medium was removed from each well of one set of samples. Cells in the wells were lysed with 0.2 mL/well RNA Lysis buffer (Promega, Durham, NC, USA). The RNA lysates were stored at − 80 °C. The cells in the second set of samples continued to culture, and the medium was changed every 3 days. On Day 7, the medium was removed from each well of the second set of samples. Cells in the wells were lysed with 0.2 mL/well RNA Lysis buffer.

### Stimulation of tenocytes with TNFα

Tenocytes at 2 × 10^4^/well were added to 48-well plates coated with CTM. After culturing for 24 h, the medium was removed. 0.5 mL/well of fresh growth medium (−) or 0.5 mL/well of fresh medium with 10 ng/mL human TNFα (+) (PeproTech, Rocky Hill, NJ) was added and cells incubated at 37 °C. After 24 h, cells were lysed with 0.2 mL/well RNA Lysis buffer. The RNA lysates were either stored at − 80 °C to be used later or used immediately for RNA isolation.

### Primers for quantitative polymerase chain reaction (qPCR)

QuantiTect primers used in qPCR were purchased from Qiagen (Germantown, MD, USA; Table 2).

**Table 2:** Primers for quantitative polymerase chain reaction. The genes and primers for qPCR are listed

Gene	Primer
Glyceraldehyde 3-Phosphate Dehydrogenase *(GAPDH)*	Hs_GAPDH_2_SG QT01192646
Scleraxis (*SCX*)	Hs_SCX_2_SG QT01529507
Tenomodulin (*TNMD*)	Hs_TNMD_1_SG QT01024590
Tenascin-C (*TNC*)	Hs_TNC_1_SG QT00024409
Type I Collagen (*COL1A1*)	Hs_COL1A1_1_SG QT00037793
Type III Collagen (*COL3A1*)	Hs_COL3A1_1_SG QT00058233
Decorin (*DCN*)	Hs_DCN_1_SG QT00032459
Tumor Necrosis Factor *(TNF)*	Hs_TNF_3_SG QT01079561
Interleukin 8 *(IL8)*	Hs_CXCL8_1_SG QT00000322
Interleukin 1 Beta *(IL-1β)*	Hs_IL1B_1_SG QT00021385
Transforming Growth Factor Beta 1 *(TFG-β1)*	Hs_TGFB1_1_SG QT00000728
Transforming Growth Factor Beta 3 (*TFG-β3)*	Hs_TGFB3_1_SG QT00001302
Matrix Metalloproteinase 1 *(MMP1)*	Hs_MMP1_1_SG QT00014581

### Quantification of the relative expression of mRNA by qPCR

The quantification of the relative expression of tenocyte phenotypic markers and inflammatory markers was performed in tenocytes cultured on CTMs by qPCR, as previously described [27]. Briefly, total RNA from cell lysates was purified using SV 96 Total RNA Isolation System (Promega, Madison, WI). RNA concentration and purity were measured using TECAN Spark Nano plate (TECAN, Switzerland). cDNA preparation and qPCR were performed on Roche Lightcycler 480, following a standard procedure. Every testing condition has 3–4 biological repeat samples, and each sample was run in duplicate. After each run was completed, a second derivative analysis was performed using the raw data to determine the mean Cp (Crossing point-PCR-cycle) for each sample. mRNA expression relative to Glyceraldehyde 3-Phosphate Dehydrogenase (*GAPDH*) [16, 55] was determined by Pfaffl analysis (2ΔCp target/2ΔCp reference), in which ΔCp = mean Cp of sample - mean Cp of the cells on Day 0 (starting cells as reference) [27].

### Statistical analysis

Each independent experiment contained 3 or more biological replicates (*n* ≥ 3), and data are presented as the mean ± standard deviation. Results shown are representative of at least two independent experiments. Analyses were conducted using IBM SPSS (Build 1.0.0.1444) and Graphpad Prism (version 9.3.1). The data were tested and found to be approximately normally distributed. One, two, and three-way analysis of variance (ANOVA) with Tukey post-hoc tests were conducted. Dependent variables included, tenocyte adhesion, tenocyte proliferation, tenocyte transwell migration (%), the maintenance of tenocyte phenotype based on the gene expression of tenocyte phenotypic markers (*SCX*, *TNC*, *COL1A1*, *COL3A1*, and *DCN*), and tenocyte inflammatory response based on the gene expression of inflammatory markers (*CXCL8*, *TGFβ1*, *TGFβ3*, and *MMP1*) at 24 h and over time. Independent variables included CTM and time. Significant interactions were evaluated with simple main effects analysis with Sidak correction for multiple comparisons. The significance level for all statistical tests was set at *p* = 0.05.

## Results

### Adhesion and proliferation of tenocytes on CTM

To evaluate the direct interaction between CTM and tenocytes, CTM particulates were coated onto ultra-low attachment plates. The optimal coating concentration of 2.6 mg/cm^2^ or 2.5 mg/well of a 48-well plate was determined by preliminary testing and previous experiments [7]. Since the ultra-low attachment surface does not support cell adhesion, cell adhesion and proliferation in the coated wells are mediated by the presence of CTM.

**Fig. 1:**
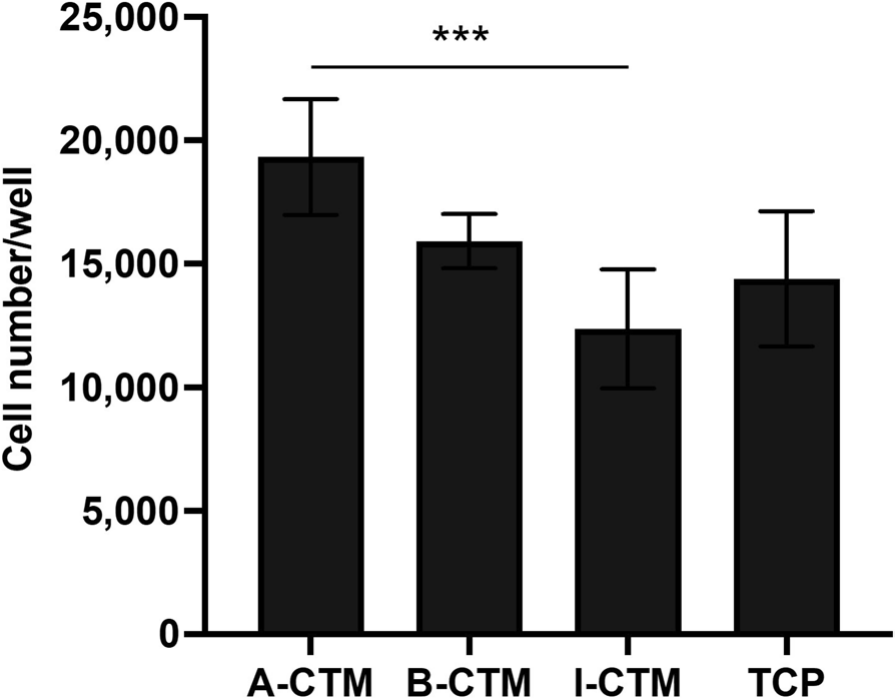
Adhesion of tenocytes on connective tissue matrices. Ultra-low attachment plate wells were coated with A-CTM, B-CTM, or I-CTM. Tenocytes were seeded onto the coated wells and incubated for 24 h. The viabilities of cells were measured by alamarBlue assay and numbers of adherent cells were converted from a standard curve. Data shown are mean ± SD (*n* = 4). *p ≤ 0.05, **p ≤ 0.01, ***p ≤ 0.005). Abbreviations: A-CTM, AmnioFill® connective tissue matrix; B-CTM, BioRenew™ connective tissue matrix; I-CTM, Interfyl® connective tissue matrix; TCP, tissue culture polystyrene

To compare the effects of different CTMs on tenocytes, the adhesion of tenocytes was evaluated on 48-well, ULAP coated with A-CTM, B-CTM, or I-CTM (*n* = 4). Tenocytes were seeded onto coated wells and incubated for 24 h. The number of adhered cells significantly varied by CTM (*p* = 0.006) with higher cell adhesion on A-CTM than I-CTM (*p* = 0.004; Fig. 1). No other significant differences between CTMs were found (*p* ≥ 0.134). These results suggest that A-CTM better supports tenocyte adhesion compared with I-CTM at 24 h.

**Fig. 2:**
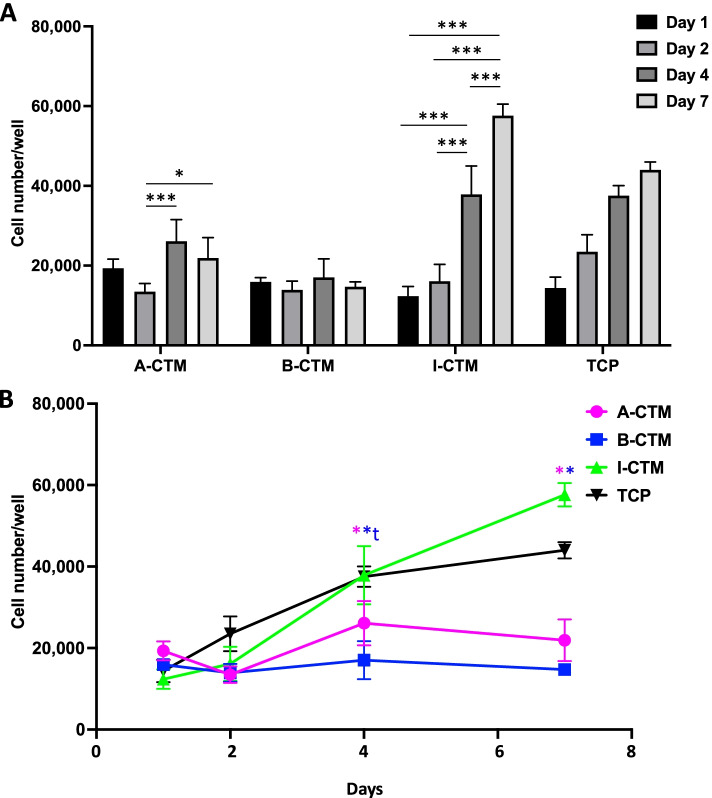
Proliferation of tenocytes on connective tissue matrices over 7 days. The viabilities of cells were measured by alamarBlue assay on Day 1, Day 2, Day 4, and Day 7 and converted to cell number, using a standard curve. The proliferation of tenocytes is compared over time for each CTM (**A**) and compared between CTMs at each time point (**B**). Data shown are mean ± SD (*n* = 4). **A** *p ≤ 0.05, **p ≤ 0.01, ***p ≤ 0.005. **B** Asterisk color indicates comparator and corresponds with legend. t *p* ≤ 0.05, compared with A-CTM, *p ≤ 0.05, compared with I-CTM. Abbreviations: A-CTM, AmnioFill® connective tissue matrix; B-CTM, BioRenew™ connective tissue matrix; I-CTM, Interfyl® connective tissue matrix; TCP, tissue culture polystyrene

The number of viable cells was monitored on Day 1, Day 2, Day 4, and Day 7. Cell number varied significantly by CTM and time (*p* < 0.001; Fig. 2; *n* = 4). Cell number was compared over time points for each CTM (Fig. 2A). The number of cells on A-CTM significantly increased from day 2 to day 4 (*p* < 0.001) and from day 2 to day 7 (*p* = 0.030). The number of cells on B-CTM remained similar across all time points (*p* ≥ 0.921). On I-CTM, the number of cells significantly increased from day 2 to day 4 (*p* < 0.001) and from day 4 to day 7 (*p* < 0.001).

Cell number was then compared between CTMs at each time point (Fig. 2B). Although there were no significant differences in the number of cells on CTMs on days 1 and 2 (*p* ≥ 0.055), on days 4 and 7, there were significantly more cells on I-CTM than on A-CTM (*p* < 0.001) and B-CTM (*p* < 0.001). In addition, on day 4, there were significantly more cells on A-CTM than B-CTM (*p* = 0.016).

To estimate the rate of tenocyte proliferation on different CTMs, growth curves were fitted using non-linear regression (Supplemental Fig. 1). The equation used was Y=Y0exp(k*X), where Y0 is cell number when X (time) is zero, and k is the rate constant. From the fitted growth curves, tenocytes showed the highest growth rate on I-CTM (0.221 cells/day), followed by A-CTM (0.046 cells/day) and B-CTM (− 0.002 cells/day). The doubling time also indicated that cells proliferated faster on I-CTM (3 days) than A-CTM (15 days) and B-CTM (no proliferation). TCP had a growth rate of 0.139 cells/day and a doubling time of 5 days.

**Fig. 3:**
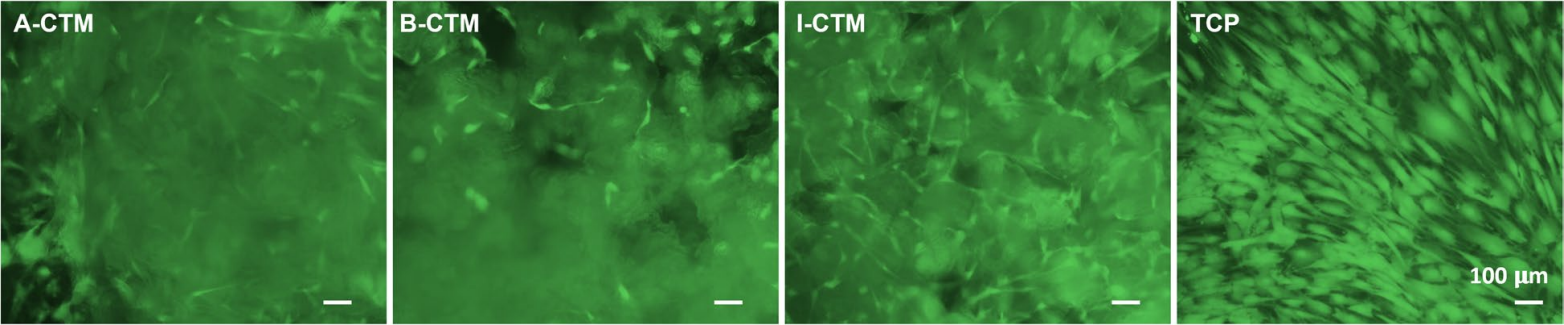
Viable tenocytes on different connective tissue matrices. Cells on different connective tissue matrices on day 7 were stained with Calcein AM. The images were captured using epi-fluorescent microscope. Representative images of each group are shown. Scale bar = 100 μm. Abbreviations: A-CTM, AmnioFill® connective tissue matrix; B-CTM, BioRenew™ connective tissue matrix; I-CTM, Interfyl® connective tissue matrix; TCP, tissue culture polystyrene

In addition, when cells were stained with Calcein AM on Day 7, viable cells detected on I-CTM looked denser than the viable cells detected on A-CTM, B-CTM or TCP (Fig. 3).

Although I-CTM demonstrated the lowest cell adhesion at 24 h, significantly lower than A-CTM and comparable to B-CTM, I-CTM promoted the greatest number of viable tenocytes over 7 days.

Among the three CTMs, significant tenocyte growth was observed on both A-CTM and I-CTM, compared to that of B-CTM. On B-CTM, tenocytes did not proliferate. For this reason, only A-CTM and I-CTM were used in the remaining experiments.

### Tenocyte migration

**Fig. 4:**
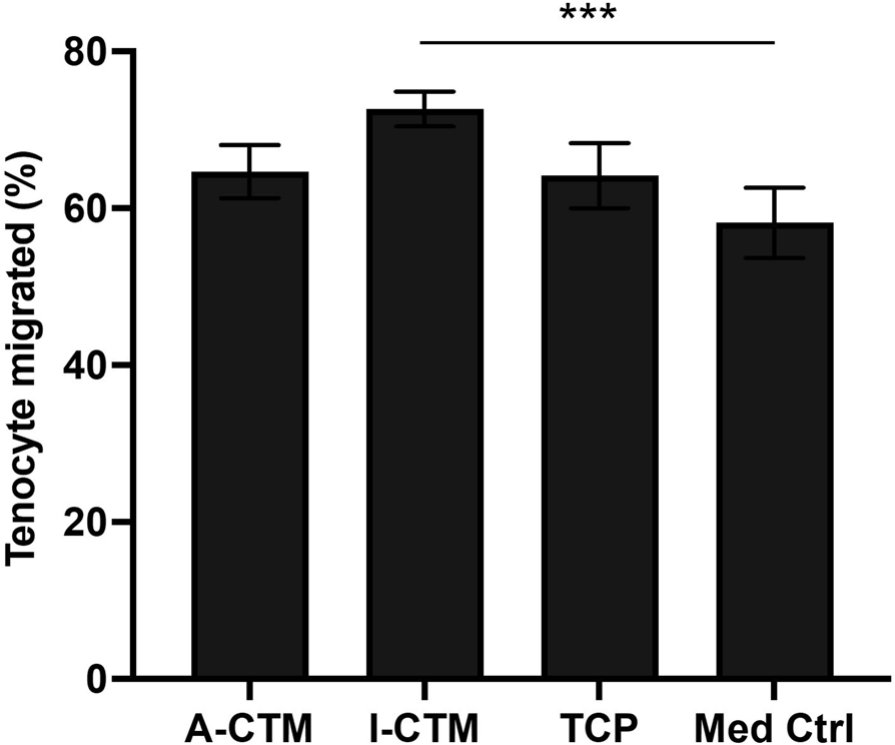
Effects of conditioned media on tenocyte migration. Conditioned medium was collected from cells cultured on connective tissue matrices for 24 h. The migration of tenocytes in the presence of conditioned media was monitored using the transwell assay. Migration was expressed as the % of migrated cells to the total number of cells. Data shown are mean ± SD (*n* = 3). *p ≤ 0.05, **p ≤ 0.01, ***p ≤ 0.005. Abbreviations: A-CTM, AmnioFill® connective tissue matrix; B-CTM, BioRenew™ connective tissue matrix; I-CTM, Interfyl® connective tissue matrix; Med Ctrl, medium control; TCP, tissue culture polystyrene

To evaluate if tenocytes cultured on different CTMs release factors that promote cell migration, conditioned medium was collected from cells cultured on different CTMs for 24 h (*n* = 3). The migration of tenocytes in the presence of conditioned media was evaluated using a transwell migration assay. A significantly higher percentage of tenocytes migrated through the transwell membrane in the presence of conditioned medium from cells on I-CTM, compared with the control medium (main effect: *p* = 0.007; post-hoc: *p* = 0.005). No other significant differences were observed between A-CTM, I-CTM, and the control medium (*p* ≥ 0.070; Fig. 4). This result suggests that tenocytes on I-CTM may release migration promoting factors into the medium and enhance the transwell migration of tenocytes.

### Phenotype maintenance

**Fig. 5:**
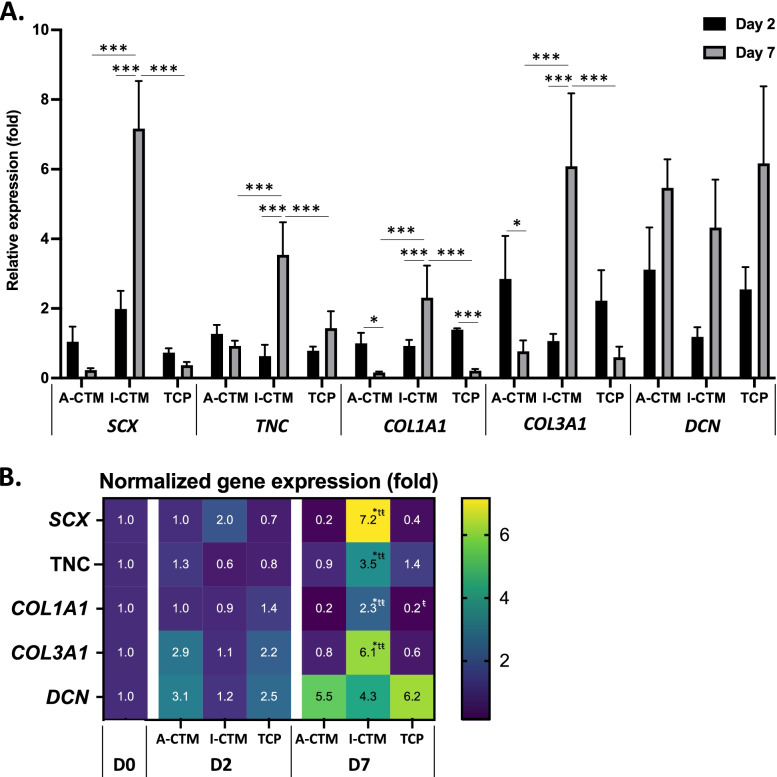
Expression of phenotypic markers in tenocytes cultured on connective tissue matrices over time. The relative expression of tenocyte phenotypic markers (i.e., *SCX*, *TNC*, *COL1A1*, *COL3A1*, *DCN*) in tenocytes cultured on A-CTM, I-CTM, and TCP for 2 and 7 days is shown. The relative expression (fold) was compared to the mRNA level in tenocytes on Day 0 (starting cells). Data are plotted as a bar graph (**A**) and as a heat map (**B**). Data shown are mean ± SD (*n* = 4 for cells on connective tissue matrices, *n* = 3 for cells on TCP). **A** *p ≤ 0.05, **p ≤ 0.01, ***p ≤ 0.005. **B** *p ≤ 0.05, compared with A-CTM; t *p* ≤ 0.05, compared with TCP; ŧ *p* ≤ 0.05, compared with day 2. Abbreviations: A-CTM, AmnioFill® connective tissue matrix; *COL1A1*, type I collagen; *COL3A1*, type III collagen; *DCN*, decorin; I-CTM, Interfyl® connective tissue matrix; *SCX*, scleraxis; TCP, tissue culture polystyrene; *TNC*, tenascin-C

To evaluate if the presence of CTM affects the dedifferentiation of tenocytes, the expressions of phenotypic markers in tenocytes cultured on A-CTM, I-CTM, or TCP for 2 days or 7 days was analyzed using qPCR (Fig. 5; *n* = 4 for cells on CTMs and *n* = 3 for cells on TCP).

In tenocytes cultured on I-CTM, the expression of *SCX* (*p* < 0.001), *TNC* (*p* < 0.001), *COL1A1* (*p* < 0.001), and *COL3A1* (*p* < 0.001) significantly increased from day 2 to day 7. Conversely, in tenocytes cultured on A-CTM and TCP, the expression of *SCX* and *TNC* remained unchanged from day 2 to day 7(*p* ≥ 0.102) and the expression of *COL1A1* significantly decreased from day 2 to day 7 (*p* ≤ 0.014). The expression of *COL31A* significantly decreased on A-CTM from day 2 to day 7 (*p* = 0.018) and remained unchanged on TCP from day 2 to day 7 (*p* = 0.094). Decorin expression increased over time for A-CTM, I-CTM, and TCP (*p* < 0.001).

**Fig. 6:**
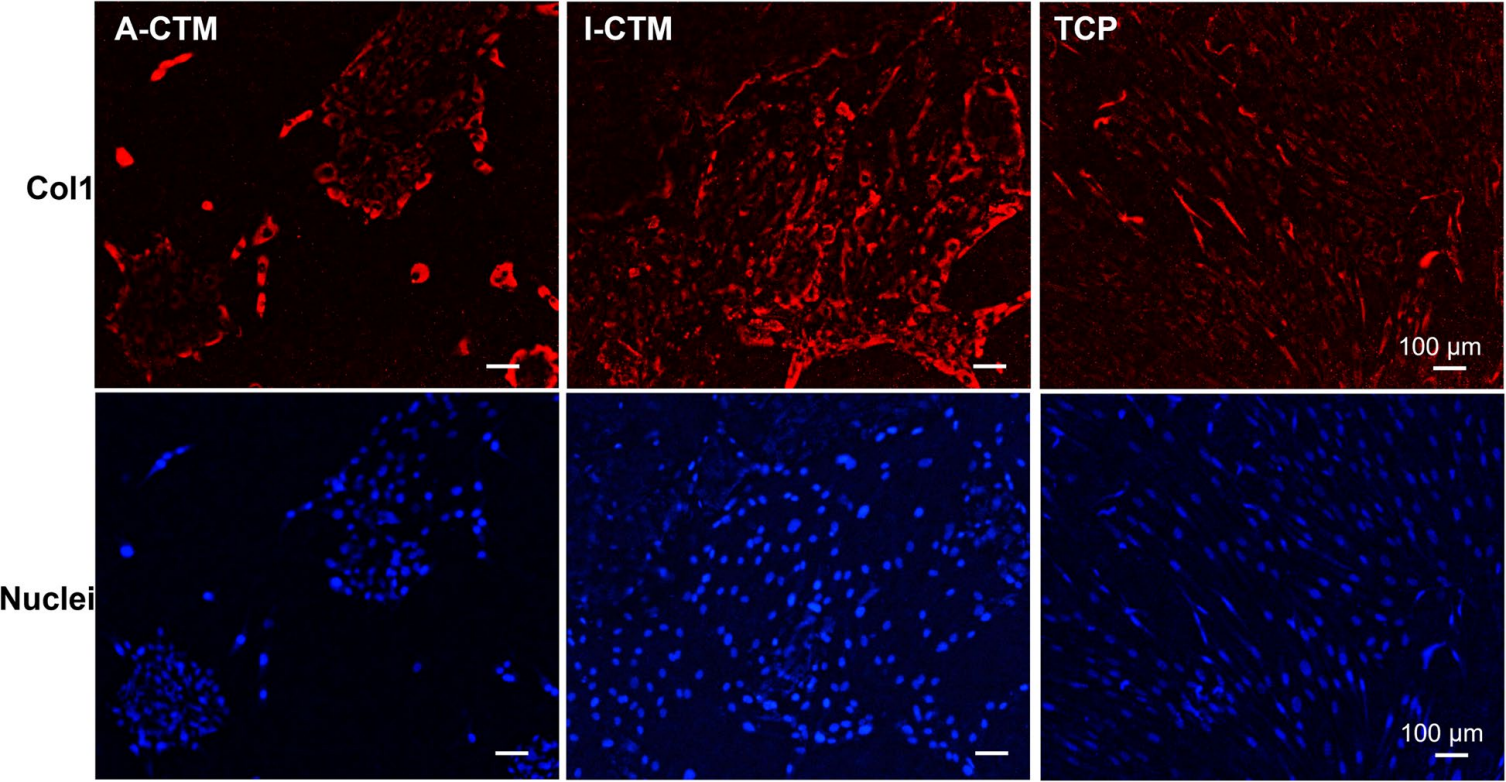
Expression of type I collagen protein in tenocytes. Tenocytes on A-CTM, I-CTM or TCP were stained with anti-Col1 (red) and Hoechst dye (blue) after cultured for 7 days. Representative images were shown. Scale bar = 100 μm. Abbreviations: A-CTM, AmnioFill® connective tissue matrix; COL1, type I collagen; I-CTM, Interfyl® connective tissue matrix; TCP, tissue culture polystyrene

As a significant increase in tenocyte phenotypic markers was seen in tenocytes cultured on I-CTM, we next evaluated type I collagen (COL1) protein expression using antibody staining and microscopy. COL1 protein expression was analyzed on tenocytes cultured on A-CTM, I-CTM, and TCP for 7 days. A more prominent expression of type I collagen was detected in cells cultured on I-CTM than on A-CTM or TCP. This is consistent with the gene expression of *COL1A1* in cells on different substrates (Fig. 6).

### Inflammatory response at 24 h

**Fig. 7:**
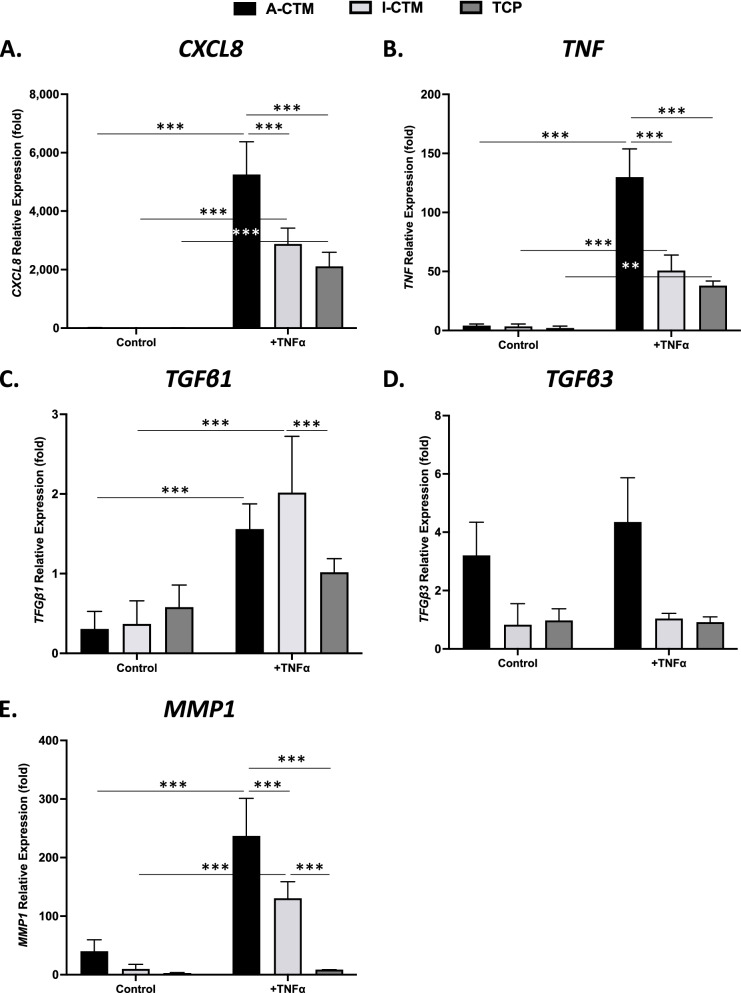
Expression of inflammatory markers in tenocytes cultured on connective tissue matrices with and without stimulation. The relative expression of inflammatory markers in tenocytes cultured on A-CTM, I-CTM, or TCP in stimulated (+TNFα) and unstimulated (Control) conditions for 24 h is shown. The relative expression (fold) of *CXCL8* (**A**), *TNF* (**B**), *TGFβ1* (**C**), *TGFβ3* (**D**), and *MMP1* (**E**) was compared to the mRNA level in tenocytes on Day 0 (starting cells). Data shown are mean ± SD (*n* = 4 for cells on CTMs, *n* = 3 for cells on TCP). *p < 0.05, **p < 0.01 and ***p < 0.005

The expressions of inflammatory markers (*CXCL8*, *TNF*, *TGFβ1*, *TGFβ3*, or *MMP1)* in stimulated (+TNF-α) and unstimulated cells (control), cultured on A-CTM, I-CTM, or TCP were analyzed by qPCR (Fig. 7; *n* = 4 for cells on CTMs and *n* = 3 for cells on TCP).

In the resting state (unstimulated condition), the expression of pro-inflammatory cytokines in tenocytes was similar between the CTMs and the control (*p* ≥ 0.398). Stimulation with TNF-α induced the expression of *CXCL8* (*p* < 0.001), *TNF* (*p* < 0.001), *TGFβ1* (*p* < 0.001), and *MMP1* (*p* < 0.001), but not *TGFβ3* (*p* < 0.306). Collapsed across stimulation states, the expression of *TGFβ3* in tenocytes on A-CTM was significantly higher than I-CTM and TCP (*p* < 0.001). With TNF-α stimulation, tenocytes cultured on A-CTM demonstrated significantly higher expressions of *CXCL8* (*p* < 0.001), *TNF* (*p* < 0.001), and *MMP1* (*p* ≤ 0.001), compared with I-CTM and TCP. Tenocytes cultured on I-CTM and on TCP demonstrated similar inflammatory profiles in the expression of *CXCL8* (*p* = 0.365), *TNF* (*p* = 0.580), and *TGFβ3* (*p* = 1.000). The expression of *TGFβ1* and *MMP1* in tenocytes, however, was significantly higher on I-CTM than TCP (*p* = 0.029).

Under inflammatory conditions, the expression of *CXCL8*, *TNF*, and *MMP1* in tenocytes was lower on I-CTM than A-CTM.

### Inflammatory response over time

**Fig. 8:**
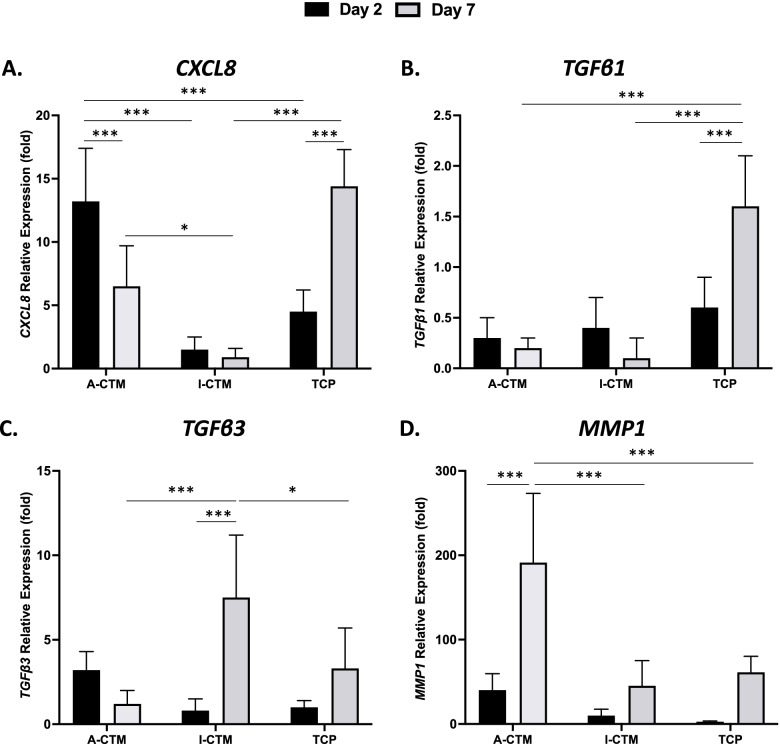
Expression of inflammatory markers in tenocytes cultured on connective tissue matrices over time. The relative expression of cytokines in tenocytes cultured on A-CTM, I-CTM, and TCP for 2 days and 7 days is shown. The relative expression (fold) of *CXCL8* (**A**), *TGFβ1* (**B**), *TGFβ3* (**C**), and *MMP1* (**D**) was compared to the mRNA level in tenocytes on Day 0 (starting cells). Data shown are mean ± SD (*n* = 4 for cells on CTMs, n = 3 for cells on TCP). *p < 0.05, **p < 0.01 and ***p < 0.005

To evaluate if CTMs have any effect on the expression of pro-inflammatory cytokines, growth factors, and proteases in tenocytes in an unstimulated state, gene expression from tenocytes cultured on A-CTM, I-CTM, and TCP for 2 days or 7 days was analyzed (Fig. 8; *n* = 4 for cells on CTMs and *n* = 3 for cells on TCP). The relative mRNA levels of *CXCL8*, *TGFβ1*, *TGFβ3*, and *MMP1* were compared between Day 2 and Day 7 and between CTMs at each time point (Fig. 8). Since the expression of *TNF* was exceptionally low in the unstimulated cells of the previous experiment (Fig. 7B), *TNF* was not included.

On day 2, there were no significant differences in the expressions of *TGFβ1*, *TGFβ3*, or *MMP1* between the CTMs and TCP (*p* ≥ 0.273). However, in tenocytes cultured on A-CTM, the relative expression of *CXCL8* was significantly higher than on I-CTM (*p* < 0.001) and TCP (*p* = 0.002).

The relative expression of *CXCL8* significantly declined over time in tenocytes cultured on A-CTM (*p* = 0.002) and TCP (*p* < 0.001). The relative expression of *TGFβ1* significantly increased over time in tenocytes cultured on TCP (*p* < 0.001). The relative expression of *TGFβ3* significantly increased over time in tenocytes cultured on I-CTM (*p* < 0.001). The relative expression of *MMP1* significantly increased over time in tenocytes cultured on A-CTM (*p* < 0.001).

On day 7, the relative expression of *CXCL8* was significantly higher on TCP than A-CTM (*p* = 0.004) and I-CTM (*p* < 0.004) and was significantly higher on A-CTM than I-CTM (*p* = 0.024). The relative expression of *TGFβ1* was significantly higher on TCP than A-CTM (*p* < 0.001) and I-CTM (*p* < 0.001). The relative expression of *TGFβ3* was significantly higher on I-CTM than on A-CTM (*p* < 0.001) and TCP (*p* = 0.033). Lastly, the relative expression of *MMP1* was significantly higher on A-CTM than on I-CTM (*p* < 0.001) and TCP (p = 0.002).

In summary, tenocytes cultured on A-CTM promoted the expression of *CXCL8* and *MMP1*, while I-CTM promoted the expression of antifibrotic growth factor *TGFβ3* over time.

## Discussion

Results from the present report demonstrate that I-CTM interacted more favorably with human tenocytes in vitro, compared with A-CTM and B-CTM. I-CTM supported tenocyte proliferation with reduced de-differentiation and attenuation of the inflammatory response. Collectively, these findings suggest that I-CTM may support tendon healing and regeneration in vivo.

Tendon healing and repair is controlled by tendon cells and their surrounding ECM [50]. It is a slow process, due to the hypovascular and hypocellular nature of tendons [24]. Connective tissue matrices have gained increasing interest for tissue engineering applications, like tendon repair, as the CTM mimics the native ECM, providing a three-dimensional structure with sites for tenocyte attachment. The interactions between the tenocytes and CTM influence several cellular processes, including tenocyte proliferation, migration, differentiation, inflammation, and finally the synthesis and organization of an ECM. Although flowable human placental CTMs represent a unique subset of scaffolds, each undergoes different processing procedures, and therefore, have different properties, which impact tenocyte-CTM interactions. The present in-vitro report examined the response of human tenocytes on three human, placental CTMs through the evaluation of tenocyte adhesion, proliferation, migration, phenotype maintenance, and inflammatory response.

Tenocyte adhesion and proliferation are two prerequisites to tendon genesis. Following tenocyte attachment to an ECM, the cells must proliferate, synthesize ECM components, and then organize ECM molecules into a functional tendon. Therefore, an increased number of tenocytes at the site of injury may improve tendon healing [24]. In examining the adhesion and proliferation of human tenocytes on three human, placental CTMs, this study found that A-CTM better supported the attachment of human tenocytes, and I-CTM supported greater tenocyte proliferation.

Initial cell adhesion to materials is often influenced by material properties, such as surface availability and roughness. However, sustained cell growth and proliferation is regulated by the interaction between the adherent cells and the physical and biochemical aspects of the CTMs. Although these analyzed CTMs all originate from human placental tissue, their properties are different due to variations in processing procedures, which may influence how tenocytes interact with the CTMs [30]. One distinct difference in tissue processing among these three CTMs is decellularization. I-CTM is decellularized to remove cellular debris and to preserve the ECM. Our finding that I-CTM supports highest tenocyte growth suggests that CTMs depleted of cellular components may provide a better substrate for tenocyte growth. The presence of bioactive growth factors and cytokines have been confirmed in A-CTM [22, 23], and B-CTM is marketed as utilizing growth factors. However, if such factors negatively affect the growth of tenocytes warrants further investigation.

No cell growth was observed on B-CTM. B-CTM is manufactured as a liquid with suspended placental CTM, whereas A-CTM and I-CTM are particulates. To make a direct comparison with A-CTM and I-CTM, the particulates in B-CTM had to be isolated from the solution and used at an equivalent concentration. In hindsight, the comparison with B-CTM is less than ideal as the product is intended to be injected in its liquid form and the process to isolate the particulates may have influenced the results. Given this limitation and the lack of tenocyte growth, B-CTM was excluded from the subsequent experiments, including tenocyte migration, tenocyte phenotype maintenance, and the inflammatory response of tenocytes.

During tendon repair, tenocytes must migrate to the site of the injury. Therefore, the effect of CTMs on tenocyte migration was evaluated. Tenocyte migration was significantly greater in the presence of conditioned medium from cells on I-CTM compared with TCP, but this was not the case for A-CTM. This finding suggests that tenocytes on I-CTM may release more migratory factors than the cells on A-CTM. A-CTM undergoes patented PURION® processing, which retains a spectrum of growth factors and cytokines [22, 23], some of which have a stimulatory effect on cell migration [22]. In contrast, I-CTM processing removes growth factors and cytokines [6]. Therefore, the factors promoting tenocyte migration in the conditioned medium from cells grown on I-CTM are most likely from the tenocytes and not I-CTM. Further experiments are needed to identify these factors.

Previous in vitro reports have demonstrated that 2D culturing of primary tenocytes leads to the dedifferentiation of tenocytes [52], and it has been hypothesized that adhesion formation post-tendon injury and repair is driven by the dedifferentiation of tenocytes [29]. Notably, however, there is evidence to suggest that ECMs can reduce the dedifferentiation of primary cells [27, 53]. To evaluate if the presence of human placental CTMs affects the dedifferentiation of tenocytes, the gene expression of tenocyte phenotype markers in tenocytes cultured on A-CTM, I-CTM, and TCP was compared using qPCR. This study found that the expression of tenocyte markers *SCX*, *TNC*, *COL1A1*, and *COL3A1* was maintained in cells cultured on I-CTM, whereas the expression of *SCX*, *TNC*, *COL1A1*, and *COL3A1* decreased over time in tenocytes cultured on A-CTM. This finding suggests that I-CTM supports the maintenance of tenocyte phenotype, whereas dedifferentiation occurred in tenocytes cultured on A-CTM.

The expression of *DCN* increased in cells on both CTMs (Fig. 5). The expression of DCN in tenocytes or tendon tissues can be modulated by cell isolation method or the rapture of tissues (prolonged elevated expression) [47, 54]. For this reason, DCN has been excluded as a tenocyte differentiation marker in some studies [16].

Although excessive inflammation is thought to impair healing, recent evidence suggests that placental-derived products can interact with tenocytes and reduce the inflammatory response to support tendon healing [30, 34]. This study sought to evaluate the effect of placental CTMs on tenocyte inflammatory gene expression (i.e., *CXCL8*, *TNF*, *TGFβ1*, *TGFβ3*, and *MMP1*). Tenocytes were evaluated in both unstimulated and stimulated states. Tenocytes were treated with TNF-α to stimulate a pro-inflammatory environment that mimics the early phase of wound healing, which resulted in increased expression of *CXCL8*, *TNF*, *TGFβ1*, and *MMP1*, but not *TGFβ3*. In the stimulated state, the pro-inflammatory response was less pronounced on I-CTM than A-CTM. Moreover, when examined over time, I-CTM promoted the gene expression of antifibrotic growth factor (*TGFβ3*) and downregulated the gene expression of a pro-inflammatory protease (*MMP1*) to a greater extent than A-CTM.

Previous research has demonstrated that the regulatory proteins contained within micronized dehydrated human amnion-chorion membrane (dHACM) reduce the expression of inflammatory genes, proteases, and ECM components, and decrease the presence of active MMPs and type III collagen [34]. The findings of the current report align with the previous report by Moreno and colleagues [34] but also suggest that the decellularization of placental tissue may promote better regulation of inflammatory processes by human tenocytes. Although decellularization is performed to reduce the host inflammatory response, it can also affect the structures and entities within the ECM, disrupting its functional characteristics [1, 5, 13]. The present work, however, suggests that the decellularization of I-CTM retains the necessary regulatory proteins and diminishes the inflammatory response of tenocytes under inflammatory conditions.

Although the effects of flowable CTM on cells have been studied previously using conditioned medium or a CTM extract [15, 20, 45], this is the first study to examine the direct interaction between tenocytes and CTMs. This distinction is important because the direct interaction between CTMs and cells better replicates the host environment for clinical application. While the present study adds to the existing literature by directly examining the interaction between tenocyte and CTMs, the study is not without limitation. Previous work suggests that there is a different response between cells derived from healthy and diseased tissue [9, 26, 33]. Only healthy cells were used in the present study. To evaluate the role of CTM in tendon repair and regeneration, the authors plan to conduct subsequent experiments to study the effects of CTM on cells isolated from diseased tissues in comparison with normal cells. Another limitation of the present report was the examination of only one cell type. In vivo, it is likely that there are interactions between the tenocytes and other cell types (e.g., macrophages), which direct the response to the scaffold. Future work may include the study of the inflammatory response of macrophages in the presence of CTM. In addition, the present study identified and evaluated inflammatory markers known to be present during the inflammatory phase of tendon healing [18, 32, 36, 48, 49]. However, there are a number of other pro-inflammatory mediators (e.g., IL6, IL1) that should be considered to provide a more comprehensive understanding of CTM inflammatory response modulation. Furthermore, the present report only used gene expression to evaluate tenocyte phenotype. Although gene expression has been used to evaluate tenocyte phenotype in other published reports [20, 34], confirming gene expression with protein levels would better elucidate the biological significance of these findings. Lastly, and perhaps most importantly, this simplified in vitro study may not predict the clinical outcomes associated with the application of these CTMs. It would be tremendously important to obtain clinical data and analyze correlations between in vitro and in vivo results.

## Conclusions

The findings from this series of experiments demonstrate significant differences in the response of tenocytes to different CTM preparations. Although A-CTM supported more tenocyte adhesion initially, I-CTM promoted tenocyte proliferation and migration. The presence of I-CTM likely prevents the loss of tenocyte phenotype, limits the expression of pro-inflammatory cytokines, growth factors, and proteases, and promotes the expression of antifibrotic growth factor *TGFβ3* to a greater extent than A-CTM. The results of this in vitro study suggest that I-CTM more favorably interacts with human tenocytes. This has significant relevance to the field as I-CTM may significantly improve tendon healing, compared with other non-decellularized CTMs. Additional investigations are needed to evaluate the clinical relevance of these findings.

## Supplementary Information

**Additional file 1: Figure 1.** Fitted growth curve of cell number over 7 days. **Table 1.** Adhesion of tenocytes. **Table 2.** Proliferation of tenocytes. **Table 3.** Migration of tenocytes. **Table 4.** Expression of phenotypic markers in tenocytes cultured on connective tissue matrices over time. **Table 5.** Expression of inflammatory markers in tenocytes cultured on connective tissue matrices with and without stimulation. **Table 6.** Expression of inflammatory markers in tenocytes cultured on connective tissue matrices across time. **Data Set 1.** Adhesion and proliferation of tenocytes. **Data Set 2.** Migration of tenocytes. **Data Set 3.** Phenotype maintenance of menocytes across time. **Data Set 4.** Inflammatory response of tenocytes with and without stimulation. **Data Set 5.** Inflammatory response of tenocytes across time.

## Data Availability

All data generated or analyzed during this study are included in this published article and its supplementary information files.
