# Supplementary material for: A decellularized flowable placental connective tissue matrix supports cellular functions of human tenocytes in vitro
Source: J Exp Orthop. 2022 Jul 18;9:69. doi: 10.1186/s40634-022-00509-4 (PMC9294091; doi:10.1186/s40634-022-00509-4)
Supplement: Supplementary file 1 — Additional file 1: Figure 1. Fitted growth curve of cell number over 7 days. Table 1. Adhesion of tenocytes. Table 2. Proliferation of tenocytes. Table 3. Migration of tenocytes. Table 4. Expression of phenotypic markers in tenocytes cultured on connective tissue matrices over time. Table 5. Expression of inflammatory markers in tenocytes cultured on connective tissue matrices with and without stimulation. Table 6. Expression of inflammatory markers in tenocytes cultured on connective tissue matrices across time. Data Set 1. Adhesion and proliferation of tenocytes. Data Set 2. Migration of tenocytes. Data Set 3. Phenotype maintenance of menocytes across time. Data Set 4. Inflammatory response of tenocytes with and without stimulation. Data Set 5. Inflammatory response of tenocytes across time. [file 40634_2022_509_MOESM1_ESM.docx]

**Supplemental Material**

**Figure 1. Fitted growth curve of cell number over 7 days.** The viabilities of cells were measured by alamarBlue assay on Day 1, Day 2, Day 4, and Day 7 and converted cell number using a standard curve. To estimate the rate of tenocyte proliferation on different CTMs, growth curves were fitted using non-linear regression. The equation used was Y=Y0exp(k*X), where Y0 is cell number when X (time) is zero, and k is the rate constant. The fitted growth curves are shown as solid black lines. Data shown are mean ± SD (n = 4). Abbreviations: A-CTM, AmnioFill® connective tissue matrix; B-CTM, BioRenewTM connective tissue matrix; I-CTM, Interfyl® connective tissue matrix; TCP, tissue culture polystyrene

**Table 1. Adhesion of tenocytes.** Ultra-low attachment plate wells were coated with A-CTM, B-CTM, or I-CTM. TCP was used as a control. Tenocytes were seeded onto the coated wells and incubated for 24 hours. The viabilities of cells were measured by alamarBlue assay and numbers of adherent cells were converted from a standard curve. Data shown are mean ± SD (n = 4).

Abbreviations: A-CTM, AmnioFill® connective tissue matrix; B-CTM, BioRenew^TM^ connective tissue matrix; I-CTM, Interfyl® connective tissue matrix; TCP, tissue culture polystyrene.

| **Connective Tissue Matrix** | **Cell Number** |
| --- | --- |
| **A-CTM** | 19,322 ± 2,345 |
| **B-CTM** | 15,919 ± 1,102 |
| **I-CTM** | 12,366 ± 2,403 |
| **TCP** | 14,384 ± 2,745 |
| **TOTAL** | **15,865 ± 3,647** |

**Table 2. Proliferation of tenocytes.** The viability of adhered cells on A-CTM, B-CTM, I-CTM, and TCP (control) was detected using alamarBlue assay at four time points (i.e., Day 1, Day 2, Day 4, and Day 7). Fluorescent intensity was expressed in arbitrary units (AU) and converted to number of cells using a standard curve. Data shown are mean ± SD (n = 4).

Abbreviations: A-CTM, AmnioFill® connective tissue matrix; B-CTM, BioRenew^TM^ connective tissue matrix; I-CTM, Interfyl® connective tissue matrix; TCP, tissue culture polystyrene.

| **Connective Tissue Matrix** | **Cell Number** | | | |
| --- | --- | --- | --- | --- |
|  | **Day 1** | **Day 2** | **Day 4** | **Day 7** |
| **A-CTM** | 19,322 ± 2,345 | 13,467 ± 2,038 | 26,109 ± 5,453 | 21,912 ± 5,104 |
| **B-CTM** | 15,919 ± 1,102 | 13,926 ± 2,211 | 17,031 ± 4,658 | 14,715 ± 1,229 |
| **I-CTM** | 12,366 ± 2,403 | 16,097 ± 4,219 | 37,855 ± 7,134 | 57,639 ± 2,879 |
| **TCP** | 14,384 ± 2,745 | 23,483 ± 4,290 | 37,551 ± 2,506 | 43,999 ± 2,007 |
| **TOTAL** | **15,470 ± 3,402** | **16,931 ± 5,213** | **30,477 ± 9,786** | **35,890 ± 17,727** |

**Table 3. Migration of tenocytes**. Conditioned medium was collected from cells cultured on connective tissue matrices for 24 hours. The migration of tenocytes in the presence of conditioned media was monitored using the transwell assay. The migration was expressed as the % of migrated cells to the total number of cells. Data shown are mean ± SD (n = 3).

Abbreviations: A-CTM, AmnioFill® connective tissue matrix; I-CTM, Interfyl® connective tissue matrix; Med Ctrl, medium control; TCP, tissue culture polystyrene.

| **Connective Tissue Matrix** | **Migration (%)** |
| --- | --- |
| **A-CTM** | 64.69 ± 3.38 |
| **I-CTM** | 72.66 ± 2.23 |
| **Med Ctrl** | 58.16 ± 4.49 |
| **TCP** | 64.19 ± 4.14 |
| **TOTAL** | **64.93 ± 6.22** |

**Table 4.** **Expression of phenotypic markers in tenocytes cultured on connective tissue matrices over time.** The relative mRNA expression of phenotype markers (i.e., *SCX*, *TNC*, *COL1A1*, *COL3A1*, *DCN*) in tenocytes were cultured on A-CTM, I-CTM, and TCP for 2 and 7 days. The relative expression (fold) was compared to the mRNA level in tenocytes on Day 0 (starting cells). Data shown are mean ± SD (n = 4 for cells on connective tissue matrices, n = 3 for cells on TCP).

Abbreviations: A-CTM, AmnioFill® connective tissue matrix; *COL1A1*, type I collagen; *COL3A1*, type III collagen; *DCN*, decorin; I-CTM, Interfyl® connective tissue matrix; *SCX*, scleraxis; TCP, tissue culture polystyrene; *TNC*, tenascin-C.

| **Gene & Connective Tissue Matrix** | **Relative mRNA Expression** | | |
| --- | --- | --- | --- |
|  | **Day 2** | **Day 7** | **TOTAL** |
| ***SCX*** |  |  |  |
| A-CTM | 1.04 ± 0.44 | 0.23 ± 0.06 | 0.64 ± 0.52 |
| I-CTM | 1.99 ± 0.52 | 7.16 ± 1.37 | 4.57 ± 2.93 |
| TCP | 0.73 ± 0.12 | 0.37 ± 0.10 | 0.55 ± 0.22 |
| ***SCX* Total** | **1.30 ± 0.67** | **2.79 ± 3.55** | **2.05 ± 2.61** |
|  |  |  |  |
| ***TNC*** |  |  |  |
| A-CTM | 1.27 ± 0.26 | 0.92 ± 0.15 | 1.10 ± 0.27 |
| I-CTM | 0.63 ± 0.32 | 3.54 ± 0.93 | 2.09 ± 1.68 |
| TCP | 0.79 ± 0.12 | 1.44 ± 0.49 | 1.11 ± 0.48 |
| ***TNC* Total** | **0.91 ± 0.38** | **2.01 ± 1.35** | **1.46 ± 1.12** |
|  |  |  |  |
| ***COL1A1*** |  |  |  |
| A-CTM | 1.00 ± 0.30 | 0.16 ± 0.03 | 0.58 ± 0.49 |
| I-CTM | 0.92 ± 0.17 | 2.31 ± 0.92 | 1.62 ± 0.96 |
| TCP | 1.39 ± 0.04 | 0.21 ± 0.05 | 0.80 ± 0.65 |
| ***COL1A1* Total** | **1.08 ± 0.28** | **0.95 ± 1.19** | **1.02 ± 0.84** |
|  |  |  |  |
| ***COL3A1*** |  |  |  |
| A-CTM | 2.85 ± 1.23 | 0.77 ± 0.31 | 1.81 ± 1.39 |
| I-CTM | 1.06 ± 0.21 | 6.08 ± 2.10 | 3.57 ± 3.02 |
| TCP | 2.22 ± 0.88 | 0.60 ± 0.30 | 1.41 ± 1.06 |
| ***COL3A1* Total** | **2.03 ± 1.13** | **2.65 ± 2.96** | **2.34 ± 2.21** |
|  |  |  |  |
| ***DCN*** |  |  |  |
| A-CTM | 3.12 ± 1.21 | 5.47 ± 0.81 | 4.29 ± 1.58 |
| I-CTM | 1.19 ± 0.27 | 4.32 ± 1.38 | 2.75 ± 1.91 |
| TCP | 2.55 ± 0.64 | 6.16 ± 2.21 | 4.36 ± 2.46 |
| ***DCN* Total** | **2.26 ± 1.15** | **5.24 ± 1.54** | **3.75 ± 2.02** |
|  |  |  |  |
| **TOTAL** | **1.52** | **2.73** | **2.12 ± 2.07** |

**Table 5.** **Expression of inflammatory markers in tenocytes cultured on connective tissue matrices with and without stimulation.** The relative mRNA expression of cytokines (i.e., *CXCL8*, *TNF*, *TGFβ*1, *TGFβ3*, *MMP1*) in tenocytes were cultured on A-CTM, I-CTM, and TCP in unstimulated (-TNFα) and stimulated (+TNFα) conditions for 24 h. The relative expression (fold) was compared to the mRNA level in tenocytes at the start of the experiment (Day 0). Data shown are mean ± SD (n = 4 for cells on CTMs, n = 3 for cells on TCP).

Abbreviations: A-CTM, AmnioFill® connective tissue matrix; B-CTM, BioRenew^TM^ connective tissue matrix; *CXCL8*, C-X-C motif chemokine ligand 8; I-CTM, Interfyl® connective tissue matrix; *MMP1*, matrix metalloproteinase 1; TCP, tissue culture polystyrene; *TGFβ1*, transforming growth factor beta 1; *TGFβ3*, transforming growth factor beta 3; *TNF*, tumor necrosis factor.

| **Gene & Connective Tissue Matrix** | **Relative mRNA Expression** | | |
| --- | --- | --- | --- |
|  | **Unstimulated** | **Stimulated** | **TOTAL** |
| ***CXCL8*** |  |  |  |
| A-CTM | 13.16 ± 4.17 | 5,254.96 ± 1,116.84 | 2,634.06 ± 2,895.69 |
| I-CTM | 1.52 ± 1.00 | 2,879.09 ± 538.70 | 1,440.31 ± 1,578.03 |
| TCP | 4.52 ± 1.68 | 2,113.40 ± 479.21 | 848.07 ± 1,179.67 |
| ***CXCL8* Total** | **6.57 ± 5.91** | **3,676.30 ± 1,571.92** | **1,754.06 ± 2,153.83** |
|  |  |  |  |
| ***TNF*** |  |  |  |
| A-CTM | 4.10 ± 1.36 | 129.98 ± 23.82 | 67.04 ± 69.07 |
| I-CTM | 3.41 ± 2.12 | 50.76 ± 13.15 | 27.09 ± 26.77 |
| TCP | 2.05 ± 1.61 | 38.02 ± 3.87 | 16.44 ± 19.83 |
| ***TNF* Total** | **3.29 ± 1.77** | **79.90 ± 46.15** | **39.77 ± 49.97** |
|  |  |  |  |
| ***TGFβ1*** |  |  |  |
| A-CTM | 0.30 ± 0.22 | 1.56 ± 0.32 | 0.93 ± 0.72 |
| I-CTM | 0.37 ± 0.29 | 2.02 ± 0.71 | 1.19 ± 1.01 |
| TCP | 0.58 ± 0.28 | 1.02 ± 0.17 | 0.75 ± 0.32 |
| ***TGFβ1* Total** | **0.40 ± 0.26** | **1.63 ± 0.60** | **0.99 ± 0.77** |
|  |  |  |  |
| ***TGFβ3*** |  |  |  |
| A-CTM | 3.21 ± 1.13 | 4.35 ± 1.52 | 3.78 ± 1.38 |
| I-CTM | 0.83 ± 0.72 | 1.04 ± 0.17 | 0.93 ± 0.50 |
| TCP | 0.97 ± 0.40 | 0.92 ± 0.18 | 0.95 ± 0.30 |
| ***TGFβ3* Total** | **1.73 ± 1.40** | **2.34 ± 1.94** | **2.02 ± 1.66** |
|  |  |  |  |
| ***MMP1*** |  |  |  |
| A-CTM | 40.11 ± 19.60 | 236.85 ± 64.18 | 138.48 ± 113.97 |
| I-CTM | 9.92 ± 7.68 | 130.52 ± 28.26 | 70.22 ± 67.25 |
| TCP | 2.71 ± 0.96 | 8.55 ± 0.07 | 5.04 ± 3.27 |
| ***MMP1* Total** | **18.93 ± 20.59** | **148.66 ± 98.00** | **80.71 ± 94.56** |
|  |  |  |  |
| **TOTAL** | **6.19 ± 11.47** | **781.76 ± 1,611.33** | **375.51 ± 1,172.54** |

**Table 6. Expression of inflammatory markers in tenocytes cultured on connective tissue matrices across time.** The relative mRNA expression of cytokines (i.e., *CXCL8*, *TGFβ1*, *TGFβ3*, *MMP1*) in tenocytes were cultured on A-CTM, I-CTM, and TCP for 2 and 7 days. The relative expression (fold) was compared to the mRNA level in tenocytes at the start of the experiment (Day 0). Abbreviations: A-CTM, AmnioFill® connective tissue matrix; B-CTM, BioRenew^TM^ connective tissue matrix; CXCL8, C-X-C motif chemokine ligand 8; I-CTM, Interfyl® connective tissue matrix; *MMP1*, matrix metalloproteinase 1; TCP, tissue culture polystyrene; *TGFβ1*, transforming growth factor beta 1; *TGFβ3*, transforming growth factor beta 3.

| **Gene & Connective Tissue Matrix** | **Relative mRNA Expression** | | |
| --- | --- | --- | --- |
|  | **Day 2** | **Day 7** | **TOTAL** |
| ***CXCL8*** |  |  |  |
| A-CTM | 13.16 ± 4.17 | 6.53 ± 3.20 | **9.84 ± 4.94** |
| I-CTM | 1.52 ± 1.00 | 0.93 ± 0.67 | **1.23 ± 0.85** |
| TCP | 4.52 ± 1.68 | 14.36 ± 2.85 | **9.44 ± 5.78** |
| ***CXCL8* Total** | **6.57 ± 5.91** | **6.63 ± 5.98** | **6.60 ± 5.80** |
|  |  |  |  |
| ***TGFβ1*** |  |  |  |
| A-CTM | 0.30 ± 0.22 | 0.16 ± 0.06 | **0.23 ± 0.17** |
| I-CTM | 0.37 ± 0.29 | 0.12 ± 0.16 | **0.24 ± 0.25** |
| TCP | 0.58 ± 0.28 | 1.55 ± 0.49 | **1.07 ± 0.64** |
| ***TGFβ1* Total** | **0.40 ± 0.26** | **0.52 ± 0.70** | **0.46 ± 0.52** |
|  |  |  |  |
| ***TGFβ3*** |  |  |  |
| A-CTM | 3.21 ± 1.13 | 1.18 ± 0.78 | **2.20 ± 1.41** |
| I-CTM | 0.83 ± 0.72 | 7.54 ± 3.67 | **4.18 ± 4.34** |
| TCP | 0.97 ± 0.40 | 3.29 ± 2.42 | **2.13 ± 2.01** |
| ***TGFβ3* Total** | **1.73 ± 1.40** | **4.07 ± 3.70** | **2.90 ± 2.98** |
|  |  |  |  |
| ***MMP1*** |  |  |  |
| A-CTM | 40.11 ± 19.60 | 191.20 ± 82.06 | **115.66 ± 97.84** |
| I-CTM | 9.92 ± 7.68 | 45.34 ± 29.88 | **27.63 ± 27.69** |
| TCP | 2.71 ± 0.96 | 61.27 ± 18.95 | **31.99 ± 34.25** |
| ***MMP1* Total** | **18.93 ± 20.59** | **102.73 ± 85.58** | **60.83 ± 74.35** |
|  |  |  |  |
| **TOTAL** | **6.91 ± 12.72** | **28.49 ± 60.00** | **17.70 ± 44.46** |

**Data Sets**

**Data Set 1. Adhesion and proliferation of tenocytes**

| **CTM** | **Time (day)** | **Cell Number** |
| --- | --- | --- |
| A-CTM | 1 | 22695.48 |
| A-CTM | 1 | 18834.61 |
| A-CTM | 1 | 18484.46 |
| A-CTM | 1 | 17277.36 |
| B-CTM | 1 | 17111.50 |
| B-CTM | 1 | 14936.87 |
| B-CTM | 1 | 15710.89 |
| B-CTM | 1 |  |
| I-CTM | 1 | 15968.90 |
| I-CTM | 1 | 11122.07 |
| I-CTM | 1 | 11297.15 |
| I-CTM | 1 | 11076.00 |
| A-CTM | 2 | 15360.74 |
| A-CTM | 2 | 13738.99 |
| A-CTM | 2 | 14181.29 |
| A-CTM | 2 | 10587.63 |
| B-CTM | 2 | 15867.54 |
| B-CTM | 2 | 11518.29 |
| B-CTM | 2 | 14393.22 |
| B-CTM | 2 |  |
| I-CTM | 2 | 22234.76 |
| I-CTM | 2 | 14466.93 |
| I-CTM | 2 | 12633.25 |
| I-CTM | 2 | 15056.66 |
| A-CTM | 4 | 32859.08 |
| A-CTM | 4 | 26630.07 |
| A-CTM | 4 | 25386.12 |
| A-CTM | 4 | 19562.55 |
| B-CTM | 4 | 21552.89 |
| B-CTM | 4 | 12246.24 |
| B-CTM | 4 | 17295.79 |
| B-CTM | 4 |  |
| I-CTM | 4 | 48330.22 |
| I-CTM | 4 | 34563.76 |
| I-CTM | 4 | 32481.28 |
| I-CTM | 4 | 36047.29 |
| A-CTM | 7 | 29329.92 |
| A-CTM | 7 | 20133.85 |
| A-CTM | 7 | 20520.86 |
| A-CTM | 7 | 17664.37 |
| B-CTM | 7 | 16079.47 |
| B-CTM | 7 | 14374.79 |
| B-CTM | 7 | 13692.92 |
| B-CTM | 7 |  |
| I-CTM | 7 | 57950.16 |
| I-CTM | 7 | 53508.77 |
| I-CTM | 7 | 59092.76 |
| I-CTM | 7 | 60004.99 |

**Data Set 2. Migration of tenocytes**

| **CTM** | **Migration (%)** |
| --- | --- |
| A-CTM | 68.52 |
| A-CTM | 63.38 |
| A-CTM | 62.16 |
| I-CTM | 72.27 |
| I-CTM | 75.07 |
| I-CTM | 70.66 |
| MedCtrl | 63.06 |
| MedCtrl | 57.18 |
| MedCtrl | 54.25 |
| TCP | 65.70 |
| TCP | 67.36 |
| TCP | 59.50 |

**Data Set 3. Phenotype maintenance of tenocytes across time**

| **CTM** | **Phenotype Marker** | **Time (day)** | **mRNA Expression** |
| --- | --- | --- | --- |
| A-CTM | *SCX* | 2 | 1.56 |
| A-CTM | *SCX* | 2 | 1.25 |
| A-CTM | *SCX* | 2 | 0.71 |
| A-CTM | *SCX* | 2 | 0.65 |
| I-CTM | *SCX* | 2 | 2.65 |
| I-CTM | *SCX* | 2 | 1.68 |
| I-CTM | *SCX* | 2 | 1.48 |
| I-CTM | *SCX* | 2 | 2.14 |
| A-CTM | *SCX* | 7 | 0.26 |
| A-CTM | *SCX* | 7 | 0.29 |
| A-CTM | *SCX* | 7 | 0.17 |
| A-CTM | *SCX* | 7 | 0.19 |
| I-CTM | *SCX* | 7 | 6.18 |
| I-CTM | *SCX* | 7 | 6.05 |
| I-CTM | *SCX* | 7 | 8.99 |
| I-CTM | *SCX* | 7 | 7.44 |
| TCP | *SCX* | 2 | 0.75 |
| TCP | *SCX* | 2 | 0.85 |
| TCP | *SCX* | 2 | 0.60 |
| TCP | *SCX* | 7 | 0.27 |
| TCP | *SCX* | 7 | 0.37 |
| TCP | *SCX* | 7 | 0.47 |
| A-CTM | *TNC* | 2 | 1.41 |
| A-CTM | *TNC* | 2 | 1.46 |
| A-CTM | *TNC* | 2 | 1.33 |
| A-CTM | *TNC* | 2 | 0.90 |
| I-CTM | *TNC* | 2 | 1.09 |
| I-CTM | *TNC* | 2 | 0.63 |
| I-CTM | *TNC* | 2 | 0.40 |
| I-CTM | *TNC* | 2 | 0.40 |
| A-CTM | *TNC* | 7 | 1.01 |
| A-CTM | *TNC* | 7 | 1.09 |
| A-CTM | *TNC* | 7 | 0.79 |
| A-CTM | *TNC* | 7 | 0.79 |
| I-CTM | *TNC* | 7 | 4.62 |
| I-CTM | *TNC* | 7 | 3.73 |
| I-CTM | *TNC* | 7 | 3.45 |
| I-CTM | *TNC* | 7 | 2.36 |
| TCP | *TNC* | 2 | 0.71 |
| TCP | *TNC* | 2 | 0.92 |
| TCP | *TNC* | 2 | 0.72 |
| TCP | *TNC* | 7 | 1.77 |
| TCP | *TNC* | 7 | 1.66 |
| TCP | *TNC* | 7 | 0.88 |
| A-CTM | *COL1A1* | 2 | 1.30 |
| A-CTM | *COL1A1* | 2 | 1.19 |
| A-CTM | *COL1A1* | 2 | 0.86 |
| A-CTM | *COL1A1* | 2 | 0.65 |
| I-CTM | *COL1A1* | 2 | 1.17 |
| I-CTM | *COL1A1* | 2 | 0.83 |
| I-CTM | *COL1A1* | 2 | 0.78 |
| I-CTM | *COL1A1* | 2 | 0.91 |
| A-CTM | *COL1A1* | 7 | 0.17 |
| A-CTM | *COL1A1* | 7 | 0.20 |
| A-CTM | *COL1A1* | 7 | 0.14 |
| A-CTM | *COL1A1* | 7 | 0.14 |
| I-CTM | *COL1A1* | 7 | 2.56 |
| I-CTM | *COL1A1* | 7 | 1.48 |
| I-CTM | *COL1A1* | 7 | 3.50 |
| I-CTM | *COL1A1* | 7 | 1.69 |
| TCP | *COL1A1* | 2 | 1.42 |
| TCP | *COL1A1* | 2 | 1.34 |
| TCP | *COL1A1* | 2 | 1.41 |
| TCP | *COL1A1* | 7 | 0.22 |
| TCP | *COL1A1* | 7 | 0.26 |
| TCP | *COL1A1* | 7 | 0.15 |
| A-CTM | *COL3A1* | 2 | 4.51 |
| A-CTM | *COL3A1* | 2 | 2.95 |
| A-CTM | *COL3A1* | 2 | 1.62 |
| A-CTM | *COL3A1* | 2 | 2.32 |
| I-CTM | *COL3A1* | 2 | 1.23 |
| I-CTM | *COL3A1* | 2 | 0.85 |
| I-CTM | *COL3A1* | 2 | 0.91 |
| I-CTM | *COL3A1* | 2 | 1.25 |
| A-CTM | *COL3A1* | 7 | 1.18 |
| A-CTM | *COL3A1* | 7 | 0.85 |
| A-CTM | *COL3A1* | 7 | 0.47 |
| A-CTM | *COL3A1* | 7 | 0.58 |
| I-CTM | *COL3A1* | 7 | 5.50 |
| I-CTM | *COL3A1* | 7 | 4.18 |
| I-CTM | *COL3A1* | 7 | 9.08 |
| I-CTM | *COL3A1* | 7 | 5.56 |
| TCP | *COL3A1* | 2 | 2.57 |
| TCP | *COL3A1* | 2 | 2.88 |
| TCP | *COL3A1* | 2 | 1.23 |
| TCP | *COL3A1* | 7 | 0.81 |
| TCP | *COL3A1* | 7 | 0.74 |
| TCP | *COL3A1* | 7 | 0.26 |
| A-CTM | *DCN* | 2 | 4.85 |
| A-CTM | *DCN* | 2 | 3.01 |
| A-CTM | *DCN* | 2 | 2.45 |
| A-CTM | *DCN* | 2 | 2.16 |
| I-CTM | *DCN* | 2 | 1.51 |
| I-CTM | *DCN* | 2 | 0.85 |
| I-CTM | *DCN* | 2 | 1.12 |
| I-CTM | *DCN* | 2 | 1.27 |
| A-CTM | *DCN* | 7 | 6.57 |
| A-CTM | *DCN* | 7 | 5.42 |
| A-CTM | *DCN* | 7 | 4.61 |
| A-CTM | *DCN* | 7 | 5.27 |
| I-CTM | *DCN* | 7 | 4.12 |
| I-CTM | *DCN* | 7 | 2.90 |
| I-CTM | *DCN* | 7 | 6.21 |
| I-CTM | *DCN* | 7 | 4.06 |
| TCP | *DCN* | 2 | 2.69 |
| TCP | *DCN* | 2 | 3.11 |
| TCP | *DCN* | 2 | 1.85 |
| TCP | *DCN* | 7 | 7.54 |
| TCP | *DCN* | 7 | 7.34 |
| TCP | *DCN* | 7 | 3.61 |

**Data Set 4. Inflammatory response of tenocytes with and without stimulation**

| **CTM** | **Stimulation Condition** | **Cytokine** | **Relative mRNA Expression** |
| --- | --- | --- | --- |
| A-CTM | Stimulated | *CXCL8* | 6212.12 |
| A-CTM | Stimulated | *CXCL8* | 5000.19 |
| A-CTM | Stimulated | *CXCL8* | 6024.98 |
| A-CTM | Stimulated | *CXCL8* | 3782.54 |
| I-CTM | Stimulated | *CXCL8* | 2806.61 |
| I-CTM | Stimulated | *CXCL8* | 3350.94 |
| I-CTM | Stimulated | *CXCL8* | 3209.95 |
| I-CTM | Stimulated | *CXCL8* | 2148.86 |
| TCP | Stimulated | *CXCL8* | 2452.25 |
| TCP | Stimulated | *CXCL8* | 1774.54 |
| A-CTM | Unstimulated | *CXCL8* | 13.55 |
| A-CTM | Unstimulated | *CXCL8* | 9.16 |
| A-CTM | Unstimulated | *CXCL8* | 11.10 |
| A-CTM | Unstimulated | *CXCL8* | 18.81 |
| I-CTM | Unstimulated | *CXCL8* | 3.01 |
| I-CTM | Unstimulated | *CXCL8* | 0.93 |
| I-CTM | Unstimulated | *CXCL8* | 0.90 |
| I-CTM | Unstimulated | *CXCL8* | 1.26 |
| TCP | Unstimulated | *CXCL8* | 6.17 |
| TCP | Unstimulated | *CXCL8* | 4.58 |
| TCP | Unstimulated | *CXCL8* | 2.82 |
| A-CTM | Stimulated | *TGFb1* | 2.00 |
| A-CTM | Stimulated | *TGFb1* | 1.58 |
| A-CTM | Stimulated | *TGFb1* | 1.29 |
| A-CTM | Stimulated | *TGFb1* | 1.36 |
| I-CTM | Stimulated | *TGFb1* | 2.61 |
| I-CTM | Stimulated | *TGFb1* | 2.34 |
| I-CTM | Stimulated | *TGFb1* | 2.10 |
| I-CTM | Stimulated | *TGFb1* | 1.00 |
| TCP | Stimulated | *TGFb1* | 1.14 |
| TCP | Stimulated | *TGFb1* | 0.90 |
| A-CTM | Unstimulated | *TGFb1* | 0.63 |
| A-CTM | Unstimulated | *TGFb1* | 0.20 |
| A-CTM | Unstimulated | *TGFb1* | 0.17 |
| A-CTM | Unstimulated | *TGFb1* | 0.21 |
| I-CTM | Unstimulated | *TGFb1* | 0.68 |
| I-CTM | Unstimulated | *TGFb1* | 0.54 |
| I-CTM | Unstimulated | *TGFb1* | 0.14 |
| I-CTM | Unstimulated | *TGFb1* | 0.10 |
| TCP | Unstimulated | *TGFb1* | 0.85 |
| TCP | Unstimulated | *TGFb1* | 0.30 |
| TCP | Unstimulated | *TGFb1* | 0.59 |
| A-CTM | Stimulated | *TGFb3* | 6.42 |
| A-CTM | Stimulated | *TGFb3* | 2.80 |
| A-CTM | Stimulated | *TGFb3* | 4.27 |
| A-CTM | Stimulated | *TGFb3* | 3.91 |
| I-CTM | Stimulated | *TGFb3* | 1.19 |
| I-CTM | Stimulated | *TGFb3* | 1.19 |
| I-CTM | Stimulated | *TGFb3* | 0.86 |
| I-CTM | Stimulated | *TGFb3* | 0.92 |
| TCP | Stimulated | *TGFb3* | 1.04 |
| TCP | Stimulated | *TGFb3* | 0.79 |
| A-CTM | Unstimulated | *TGFb3* | 4.14 |
| A-CTM | Unstimulated | *TGFb3* | 2.10 |
| A-CTM | Unstimulated | *TGFb3* | 2.37 |
| A-CTM | Unstimulated | *TGFb3* | 4.23 |
| I-CTM | Unstimulated | *TGFb3* | 1.82 |
| I-CTM | Unstimulated | *TGFb3* | 0.88 |
| I-CTM | Unstimulated | *TGFb3* | 0.22 |
| I-CTM | Unstimulated | *TGFb3* | 0.39 |
| TCP | Unstimulated | *TGFb3* | 1.10 |
| TCP | Unstimulated | *TGFb3* | 0.52 |
| TCP | Unstimulated | *TGFb3* | 1.29 |
| A-CTM | Stimulated | *MMP1* | 297.83 |
| A-CTM | Stimulated | *MMP1* | 147.87 |
| A-CTM | Stimulated | *MMP1* | 263.65 |
| A-CTM | Stimulated | *MMP1* | 238.04 |
| I-CTM | Stimulated | *MMP1* | 121.54 |
| I-CTM | Stimulated | *MMP1* | 145.07 |
| I-CTM | Stimulated | *MMP1* | 160.06 |
| I-CTM | Stimulated | *MMP1* | 95.42 |
| TCP | Stimulated | *MMP1* | 8.49 |
| TCP | Stimulated | *MMP1* | 8.60 |
| A-CTM | Unstimulated | *MMP1* | 34.87 |
| A-CTM | Unstimulated | *MMP1* | 18.82 |
| A-CTM | Unstimulated | *MMP1* | 66.02 |
| A-CTM | Unstimulated | *MMP1* | 40.74 |
| I-CTM | Unstimulated | *MMP1* | 21.03 |
| I-CTM | Unstimulated | *MMP1* | 9.04 |
| I-CTM | Unstimulated | *MMP1* | 4.43 |
| I-CTM | Unstimulated | *MMP1* | 5.18 |
| TCP | Unstimulated | *MMP1* | 2.30 |
| TCP | Unstimulated | *MMP1* | 3.81 |
| TCP | Unstimulated | *MMP1* | 2.02 |
| A-CTM | Stimulated | *TNF* | 159.81 |
| A-CTM | Stimulated | *TNF* | 127.19 |
| A-CTM | Stimulated | *TNF* | 131.26 |
| A-CTM | Stimulated | *TNF* | 101.64 |
| I-CTM | Stimulated | *TNF* | 57.03 |
| I-CTM | Stimulated | *TNF* | 31.40 |
| I-CTM | Stimulated | *TNF* | 54.18 |
| I-CTM | Stimulated | *TNF* | 60.42 |
| TCP | Stimulated | *TNF* | 35.29 |
| TCP | Stimulated | *TNF* | 40.76 |
| A-CTM | Unstimulated | *TNF* | 4.07 |
| A-CTM | Unstimulated | *TNF* | 2.19 |
| A-CTM | Unstimulated | *TNF* | 5.20 |
| A-CTM | Unstimulated | *TNF* | 4.95 |
| I-CTM | Unstimulated | *TNF* | 0.69 |
| I-CTM | Unstimulated | *TNF* | 4.24 |
| I-CTM | Unstimulated | *TNF* | 5.69 |
| I-CTM | Unstimulated | *TNF* | 3.03 |
| TCP | Unstimulated | *TNF* | 3.77 |
| TCP | Unstimulated | *TNF* | 1.80 |
| TCP | Unstimulated | *TNF* | 0.59 |

**Data Set 5. Inflammatory response of tenocytes across time**

| **CTM** | **Time (Day)** | **Gene** | **Relative mRNA Expression** |
| --- | --- | --- | --- |
| A-CTM | 2 | *CXCL8* | 13.55 |
| A-CTM | 2 | *CXCL8* | 9.16 |
| A-CTM | 2 | *CXCL8* | 11.10 |
| A-CTM | 2 | *CXCL8* | 18.81 |
| I-CTM | 2 | *CXCL8* | 3.01 |
| I-CTM | 2 | *CXCL8* | 0.93 |
| I-CTM | 2 | *CXCL8* | 0.90 |
| I-CTM | 2 | *CXCL8* | 1.26 |
| A-CTM | 7 | *CXCL8* | 10.97 |
| A-CTM | 7 | *CXCL8* | 6.77 |
| A-CTM | 7 | *CXCL8* | 3.99 |
| A-CTM | 7 | *CXCL8* | 4.40 |
| I-CTM | 7 | *CXCL8* | 0.22 |
| I-CTM | 7 | *CXCL8* | 1.35 |
| I-CTM | 7 | *CXCL8* | 1.62 |
| I-CTM | 7 | *CXCL8* | 0.51 |
| TCP | 2 | *CXCL8* | 6.17 |
| TCP | 2 | *CXCL8* | 4.58 |
| TCP | 2 | *CXCL8* | 2.82 |
| TCP | 7 | *CXCL8* | 16.41 |
| TCP | 7 | *CXCL8* | 15.58 |
| TCP | 7 | *CXCL8* | 11.10 |
| A-CTM | 2 | *TGFb1* | 0.63 |
| A-CTM | 2 | *TGFb1* | 0.20 |
| A-CTM | 2 | *TGFb1* | 0.17 |
| A-CTM | 2 | *TGFb1* | 0.21 |
| I-CTM | 2 | *TGFb1* | 0.68 |
| I-CTM | 2 | *TGFb1* | 0.54 |
| I-CTM | 2 | *TGFb1* | 0.14 |
| I-CTM | 2 | *TGFb1* | 0.10 |
| A-CTM | 7 | *TGFb1* | 0.24 |
| A-CTM | 7 | *TGFb1* | 0.16 |
| A-CTM | 7 | *TGFb1* | 0.10 |
| A-CTM | 7 | *TGFb1* | 0.13 |
| I-CTM | 7 | *TGFb1* | 0.35 |
| I-CTM | 7 | *TGFb1* | 0.02 |
| I-CTM | 7 | *TGFb1* | 0.08 |
| I-CTM | 7 | *TGFb1* | 0.01 |
| TCP | 2 | *TGFb1* | 0.85 |
| TCP | 2 | *TGFb1* | 0.30 |
| TCP | 2 | *TGFb1* | 0.59 |
| TCP | 7 | *TGFb1* | 1.08 |
| TCP | 7 | *TGFb1* | 1.53 |
| TCP | 7 | *TGFb1* | 2.06 |
| A-CTM | 2 | *TGFb3* | 4.14 |
| A-CTM | 2 | *TGFb3* | 2.10 |
| A-CTM | 2 | *TGFb3* | 2.37 |
| A-CTM | 2 | *TGFb3* | 4.23 |
| I-CTM | 2 | *TGFb3* | 1.82 |
| I-CTM | 2 | *TGFb3* | 0.88 |
| I-CTM | 2 | *TGFb3* | 0.22 |
| I-CTM | 2 | *TGFb3* | 0.39 |
| A-CTM | 7 | *TGFb3* | 2.33 |
| A-CTM | 7 | *TGFb3* | 0.93 |
| A-CTM | 7 | *TGFb3* | 0.56 |
| A-CTM | 7 | *TGFb3* | 0.92 |
| I-CTM | 7 | *TGFb3* | 5.19 |
| I-CTM | 7 | *TGFb3* | 8.36 |
| I-CTM | 7 | *TGFb3* | 12.37 |
| I-CTM | 7 | *TGFb3* | 4.23 |
| TCP | 2 | *TGFb3* | 1.10 |
| TCP | 2 | *TGFb3* | 0.52 |
| TCP | 2 | *TGFb3* | 1.29 |
| TCP | 7 | *TGFb3* | 0.49 |
| TCP | 7 | *TGFb3* | 4.63 |
| TCP | 7 | *TGFb3* | 4.75 |
| A-CTM | 2 | *MMP1* | 34.87 |
| A-CTM | 2 | *MMP1* | 18.82 |
| A-CTM | 2 | *MMP1* | 66.02 |
| A-CTM | 2 | *MMP1* | 40.74 |
| I-CTM | 2 | *MMP1* | 21.03 |
| I-CTM | 2 | *MMP1* | 9.04 |
| I-CTM | 2 | *MMP1* | 4.43 |
| I-CTM | 2 | *MMP1* | 5.18 |
| A-CTM | 7 | *MMP1* | 232.77 |
| A-CTM | 7 | *MMP1* | 284.74 |
| A-CTM | 7 | *MMP1* | 141.00 |
| A-CTM | 7 | *MMP1* | 106.31 |
| I-CTM | 7 | *MMP1* | 19.41 |
| I-CTM | 7 | *MMP1* | 34.81 |
| I-CTM | 7 | *MMP1* | 88.38 |
| I-CTM | 7 | *MMP1* | 38.77 |
| TCP | 2 | *MMP1* | 2.30 |
| TCP | 2 | *MMP1* | 3.81 |
| TCP | 2 | *MMP1* | 2.02 |
| TCP | 7 | *MMP1* | 62.68 |
| TCP | 7 | *MMP1* | 79.48 |
| TCP | 7 | *MMP1* | 41.66 |
